# Supplementary material for: Predicting visual function by interpreting a neuronal wiring diagram
Source: Nature. 2024 Oct 2;634(8032):113–23. doi: 10.1038/s41586-024-07953-5 (PMC11446822; doi:10.1038/s41586-024-07953-5)
Supplement: Supplementary file 6 — CRF and ERF predictions for individual TmY4 and TmY9 cells. Analogous to Supplementary Data 3, but for TmY target types. Shown are the top four monosynaptic pathways, the strongest pathway passing through each of the top ten intermediary types (ranking from Extended Data Fig. 7), and the trisynaptic pathway Tm1–TmY–Dm3–TmY (see the section entitled Prediction of spatial normalization). [file 41586_2024_7953_MOESM6_ESM.zip › DataS4/TmY9q/720575940630391490.pdf]

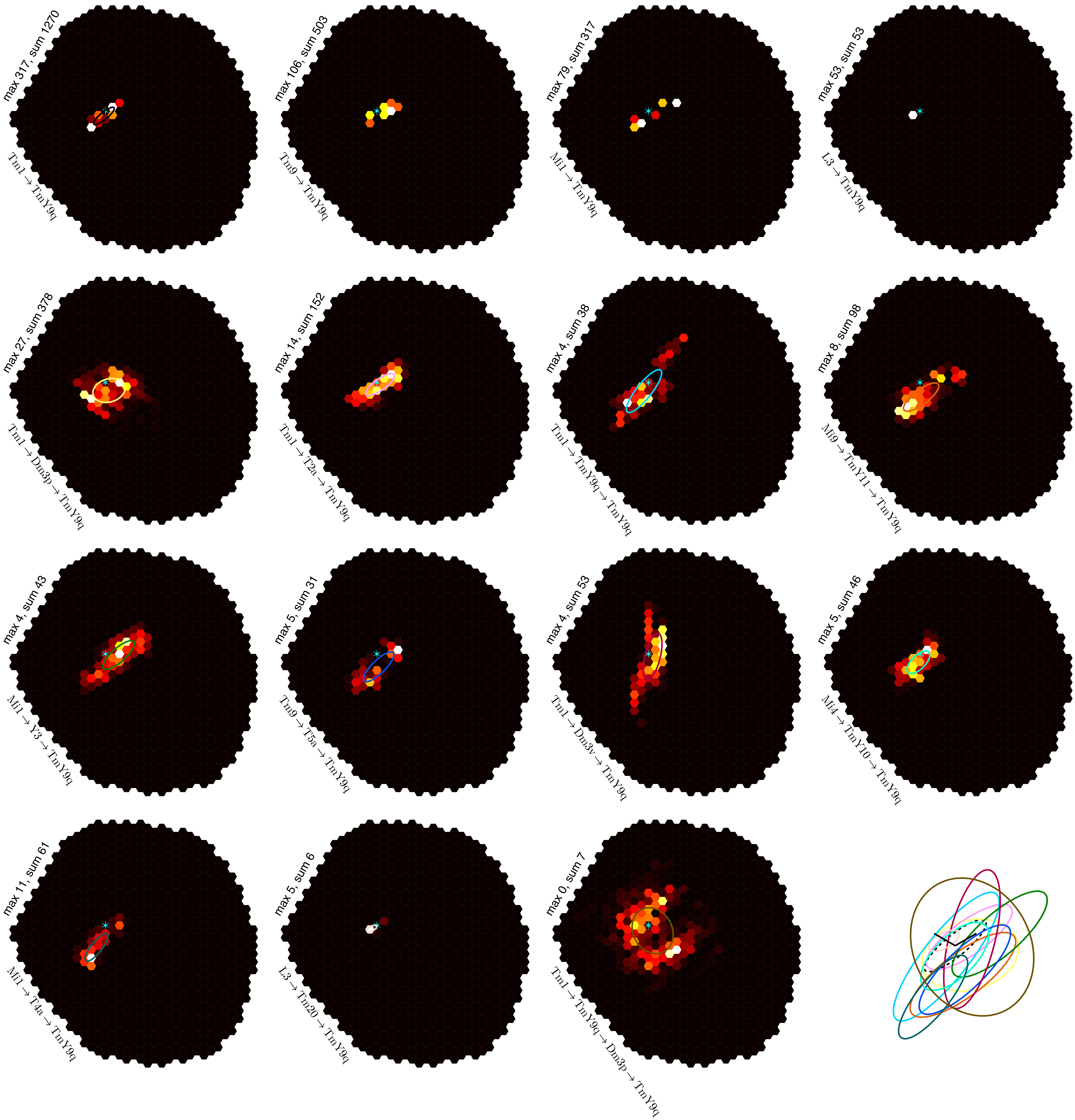
max 317, sum 1270
Tm1 → TmY9q
max 106, sum 503
Tm9 → TmY9q
max 79, sum 317
Mi1 → TmY9q
max 53, sum 53
L3 → TmY9q
max 27, sum 378
Tm1 → Dm3p → TmY9q
max 14, sum 152
Tm1 → T2a → TmY9q
max 4, sum 38
Tm1 → TmY9q → TmY9q
max 8, sum 98
Mi9 → TmY11 → TmY9q
max 4, sum 43
Mi1 → Y3 → TmY9q
max 5, sum 31
Tm9 → T5a → TmY9q
max 4, sum 53
Tm1 → Dm3v → TmY9q
max 5, sum 46
Mi4 → TmY10 → TmY9q
max 11, sum 61
Mi1 → T4a → TmY9q
max 5, sum 6
L3 → Tm20 → TmY9q
max 0, sum 7
Tm1 → TmY9q → Dm3p → TmY9q
